# Synchrotron-radiation-stimulated etching of polydimethylsiloxane using XeF_2_ as a reaction gas

**DOI:** 10.1107/S0909049509045658

**Published:** 2009-11-26

**Authors:** Tsung-Yi Chiang, Tetsuya Makimura, Tingchao He, Shuichi Torii, Tomoko Yoshida, Ryugo Tero, Changshun Wang, Tsuneo Urisu

**Affiliations:** a Institute for Molecular Science, Okazaki 444-8585, Japan; b Institute of Applied Physics, University of Tsukuba, Tsukuba 305-8573, Japan; c Department of Physics, Shanghai Jiao Tong University, Shanghai 200240, People’s Republic of China; d Graduate School of Engineering, Nagoya University, Furo-cho, Chikusa-ku, Nagoya 464-8603, Japan; e Graduate University for Advanced Studies, Okazaki 444-8585, Japan

**Keywords:** PDMS, synchrotron radiation etching, XeF_2_, anisotropy

## Abstract

The synchrotron radiation (SR) stimulated etching of silicon elastomer polydimethylsiloxane (PDMS) using XeF_2_ as an etching gas has been demonstrated. An etching system with differential pumps and two parabolic focusing mirrors was constructed to perform the etching. The PDMS was found to be effectively etched by the SR irradiation under the XeF_2_ gas flow, and the etching process was area-selective and anisotropic. An extremely high etching rate of 40–50 µm (10 min)^−1^ was easily obtained at an XeF_2_ gas pressure of 0.2–0.4 torr. This suggests that SR etching using XeF_2_ gas provides a new microfabrication technology for thick PDMS membranes, which can open new applications such as the formation of three-dimensional microfluidic circuits.

## Abstract 2

Synchrotron-radiation-stimulated etching of silicon elastomer polydimethylsiloxane using XeF_2_ as an etching gas is demonstrated.

## 1. Introduction

Poly(dimethylsiloxane) (PDMS) elastomer is used in many applications including microfluidic circuits (Jo *et al.*, 2000 ▶; Taylor *et al.*, 2005 ▶), insulation, micro/nanoelectromechanical devices (Li *et al.*, 2006 ▶) and soft lithography (Xia & Whitsides, 1998 ▶). PDMS is also biocompatible, effective for fluid seals, and transparent allowing optical alignment. The ability to reliably pattern PDMS in the form of both thick substrates and thin membranes or films is critical to expand the scope of its applications. The most popular patterning method is the molding method (Folch *et al.*, 1999 ▶), and recently many efforts to realise a more fine patterning have been reported (Xia & Whitsides, 1998 ▶). The molded PDMS membrane was used as a stamp to pattern proteins on solid substrates for the subsequent patterning of mammalian cells (James *et al.*, 1998 ▶). The other PDMS-patterning method is ‘removing-type’ patterning.

It was attempted to pattern PDMS by both wet chemical etching and dry (plasma) etching (Garra *et al.*, 2002 ▶). Compared with the wet etching method, the dry etching process has the advantages of a more stable etching rate and much higher anisotropy. However, this technique is still under development, since the patterned PDMS has a very high surface roughness that likely prevents its use for some applications. It is also difficult to pattern PDMS directly with photolithography owing to its high elongation ratio and mechanical compliance properties (Harkness *et al.*, 2004 ▶). The recently reported bond-detach lithography method successfully demonstrated nano-size patterning (Thangawng *et al.*, 2007 ▶). All these removable-type patterning techniques of PDMS are, however, applicable only to very thin PDMS membranes. A conventionally used removable technique for thick PDMS materials is mechanical drilling or punching. The advantage of the machining is its high reliability. However, the possible minimum size formed by this technique is limited to several hundreds of micrometres owing to the elasticity of PDMS. Thus we think that the development of reliable removing-type microfabrication techniques applicable to thick PDMS membranes is an important issue and will realise new three-dimensional microstructures of PDMS by combination with the molding-type techniques.

The synchrotron radiation (SR) stimulated etching process (Urisu & Kyuragi, 1987 ▶; Streller *et al.*, 1998 ▶; Akazawa *et al.*, 1991 ▶; Wen & Chou, 2000 ▶; Nonogaki *et al.*, 2000 ▶; Wang *et al.*, 2006 ▶) has many unique features, such as selective cleaving of chemical bonds by exciting certain dissociative energy levels, low damage to substrates in comparison with the plasma process, high material selectivity, anisotropic etching, lowering of the process temperature, and high spatial resolution and aspect ratio because of the short wavelengths. Although a large number of studies have been reported on SR etching on solid materials, there are no studies reporting the SR process on elastic materials to our knowledge.

In the study of SR etching of SiO_2_ substrates using XeF_2_, Streller *et al.* (1998 ▶) reported that surface excitation is dominant and that area-selectivity, anisotropy, high quantum efficiency and high spatial resolution can be achieved. Although SR etching of PDMS using XeF_2_ has not yet been reported, it is strongly expected that similar high-speed, high-spatial-resolution and high-aspect-ratio etching can be realised by this technique, since PDMS is a silicon-based polymer.

In the present work, we have constructed a new SR etching beamline consisting of differential pumping and an etching chamber with a parabolic mirror which focuses the SR beam to a pinhole which realises effective differential pumping. An extremely high speed of PDMS etching, 40–50 µm (10 min)^−1^, which suggests a new removable-type thick membrane microfabrication of PDMS, has been obtained at an XeF_2_ pressure of 0.2–0.4 torr in an etching chamber. The etching mechanism of PDMS, however, seems to be slightly different from that of SiO_2_. Insufficient pumping of the etching chamber caused the deposition of hydrogen-containing carbon material (C_*x*_H_*y*_), which often severely hindered the etching rate. This may be because PDMS has carbon as one of its major components.

## 2. Experiment

### 2.1. Materials

XeF_2_ (5 g) sealed in an SUS cylinder was purchased from Trichemical Laboratories and attached to the gas-supplying system directly. Gold-coated Cu gaskets and Ni gaskets (Ailin Vacuum) were used to connect the etching chamber, focusing mirror chamber and gas-supplying system. All parts of the end-station were made of stainless steel, except the pressure gauges which use inconel diaphragms.

The PDMS substrates used in the experiments were fabricated from a 10:1 (weight ratio) mixture of PDMS Sylgard Silicone Elastomer 184W/C and Sylgard Curing Agent 184 (Dow Corning). The solution in a template was left in-vacuum for about 30 min to remove gas bubbles. Subsequently the gelled PDMS was cured at 313 K for 20 h, and then cooled to room temperature. The solidified PDMS was carefully peeled from the template and cut to a size of 7 mm × 14 mm. Etching was carried out by SR beam irradiation either directly or through a copper mesh with 400 wires per inch (Veco 0400-Cu, Okenshoji) on the PDMS substrate surface under the XeF_2_ gas flow.

### 2.2. Beamline and etching chamber

**Figure 1:**
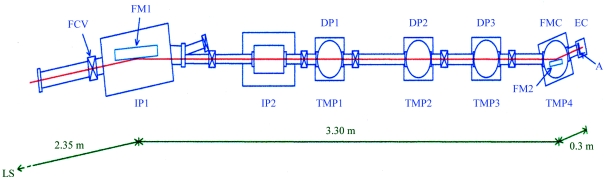
Schematic structure of the SR XeF_2_ etching beamline at UVSOR. LS: light source; FCV: fast-closing valve; FM1, FM2: first and second focusing mirrors; FMC: second focusing mirror chamber; DP*i* (*i* = 1–3): differential pumping chambers; IP*i* (*i* = 1–2): ion pumps; TMP*i* (*i* = 1–4): turbomolecular pumps; EC: etching chamber; A: aperture. Distances from the light source are shown in the figure. The vacuum pressures under usual operations were ∼10^−10^ torr at the FM1 chamber and ∼10^−9^, ∼10^−8^, ∼10^−7^ and ∼10^−4^ torr at DP1, DP2, DP3 and FMC, respectively.

**Figure 2:**
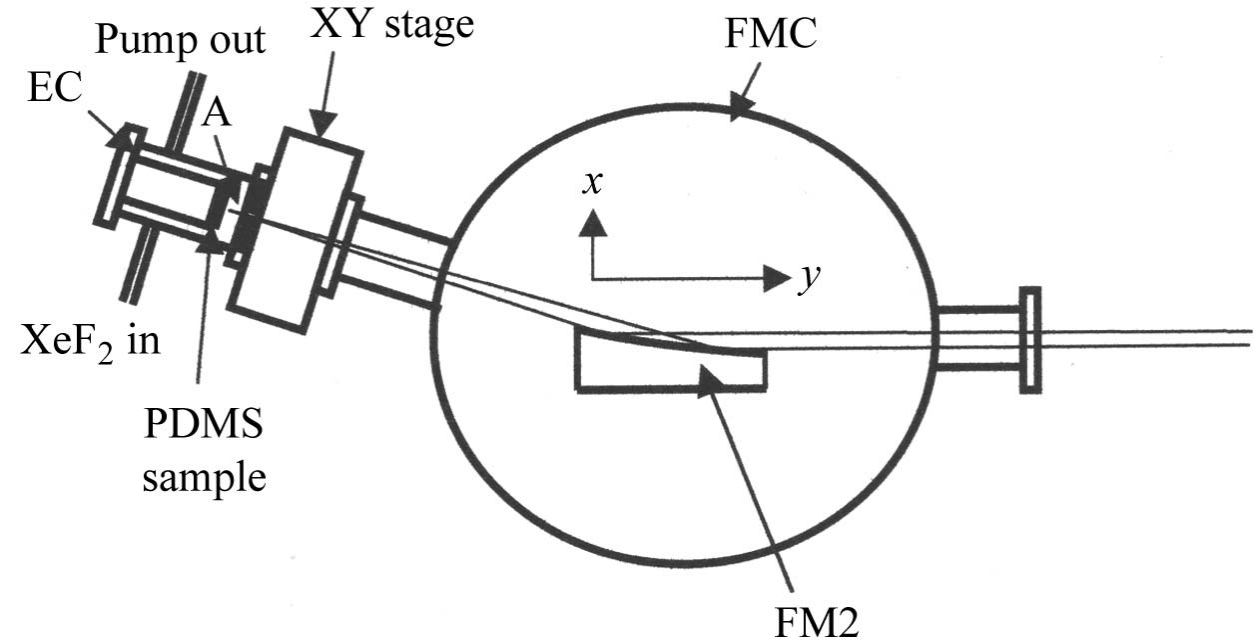
Schematic view of the beamline end-station consisting of the etching chamber (EC) and the focusing mirror chamber (FMC). FM2: second focusing mirror [parabolic, *y* = *x* ^2^(4*f*)^−1^] of length 234.7 mm and focal length *f* = 2 mm. The distance between the mirror centre and the entrance of the aperture (A) is 360 mm. The mirror positions *x*, *y* and *z* and the rotation around the *z* axis are controlled manually. The position of the aperture is changed by the XY stage. The grazing-incidence angle of SR to the mirror is 5°.

The SR etching of PDMS substrate was carried out at beamline 4A1 of the SR facility (UVSOR) at the Institute for Molecular Science (Okazaki, Japan). The beamline structure is schematically illustrated in Fig. 1 ▶. The end-station consists of a parabolic focusing mirror chamber (FMC) and an etching chamber (EC) as shown in Fig. 2 ▶. The SR beam from the light source is focused by a Pt-coated vertical pre-mirror (FM1 in Fig. 1 ▶, grazing incident angle 4°) and precisely focused by the Au-coated vertical parabolic mirror (FM2 in Figs. 1 ▶ and 2 ▶, grazing incident angle 5°) to an aperture with 1 mm diameter and 10 mm length, then irradiates on the surface of the PDMS sample in the etching chamber (Fig. 2 ▶). This aperture was effective in keeping a sufficiently high vacuum (<10^−5^ torr) of the focusing mirror chamber during the etching reaction where gas pressure in the etching chamber increased to about 1 torr. The vacuum pressures under usual operations were ∼10^−10^ torr at the FM1 chamber and ∼10^−9^, ∼10^−8^, ∼10^−7^ and ∼10^−4^ torr at DP1, DP2, DP3 and FMC, respectively.

The distance between the FM2 mirror centre and the sample is about 300 mm. The SR beam spot size on the sample surface was about 0.5 mm in diameter. The etching chamber is connected to the focusing mirror chamber through an XY stage with bellows so that the aperture position can be adjusted to the focused SR beam position. The calculated photon flux was 4 × 10^16^ photons s^−1^ at 100 mA ring current. Precise estimation of the effective photon flux is, however, difficult owing to the reflectivity degradations of the FM1 and FM2 mirrors by contamination with residual hydrocarbon gases. Therefore the relative photon flux was roughly estimated in each experiment by measuring the photoemission current of the Pt plate set at the sample position.

### 2.3. Control of the XeF_2_ gas pressure

**Figure 3:**
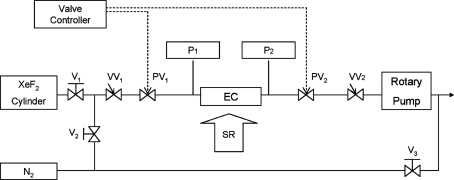
Schematic diagram of the gas-supplying system. P1, P2: diaphragm pressure gauges; VV1, VV2: variable leak valves; PV1, PV2: normally closed air actuator valves; V1, V2, V3: stop valves. The gas line and the etching chamber are purged by nitrogen gas.

Since XeF_2_ gas strongly reacts on the wall of the container and this makes the pressure inside the reaction chamber unstable, XeF_2_ is usually supplied to the reaction chamber by pulse injection and evacuation (Chu *et al.*, 1997 ▶; Sugano & Tabata, 2002 ▶). Fig. 3 ▶ shows a schematic diagram of the XeF_2_ gas-supplying system used in this experiment. The etching chamber was evacuated using a turbomolecular pump and a rotary pump. The exhaust gas was diluted with N_2_ gas and sent to a ventilation duct *via* an adsorption column. The gas pressure of the etching chamber was monitored by the inconel diaphragm pressure gauges P1 and P2 (Fig. 3 ▶).

The XeF_2_ gas pressure in the SUS cylinder was at gas–solid phase thermal equilibrium (∼4 torr). The reaction gas (100% XeF_2_) pressure was controlled in the range 0.1–0.5 torr in the etching chamber by a repeating open–close sequence of the valves PV_1_ and PV_2_ in Fig. 3 ▶, which were computer-controlled with two timers.

In this pressure range, a pressure difference of about 10^4^ is realised between the etching chamber and the FM2 mirror chamber when the 1 mm-diameter aperture was used. After etching, both the etching chamber and the FM2 mirror chamber are continuously pumped and purged with N_2_ gas flow.

## 3. Results and discussion

First, we have measured the etching characteristics for the direct SR beam irradiation of the PDMS sample surface without mask. The surface structure of the sample after etching was observed with an optical-microscopy, non-contact three-dimensional profile meter (NH-3SP, Mitakakohki) and scanning electron microscope (JSM-6700F, Jeol).

**Figure 4:**
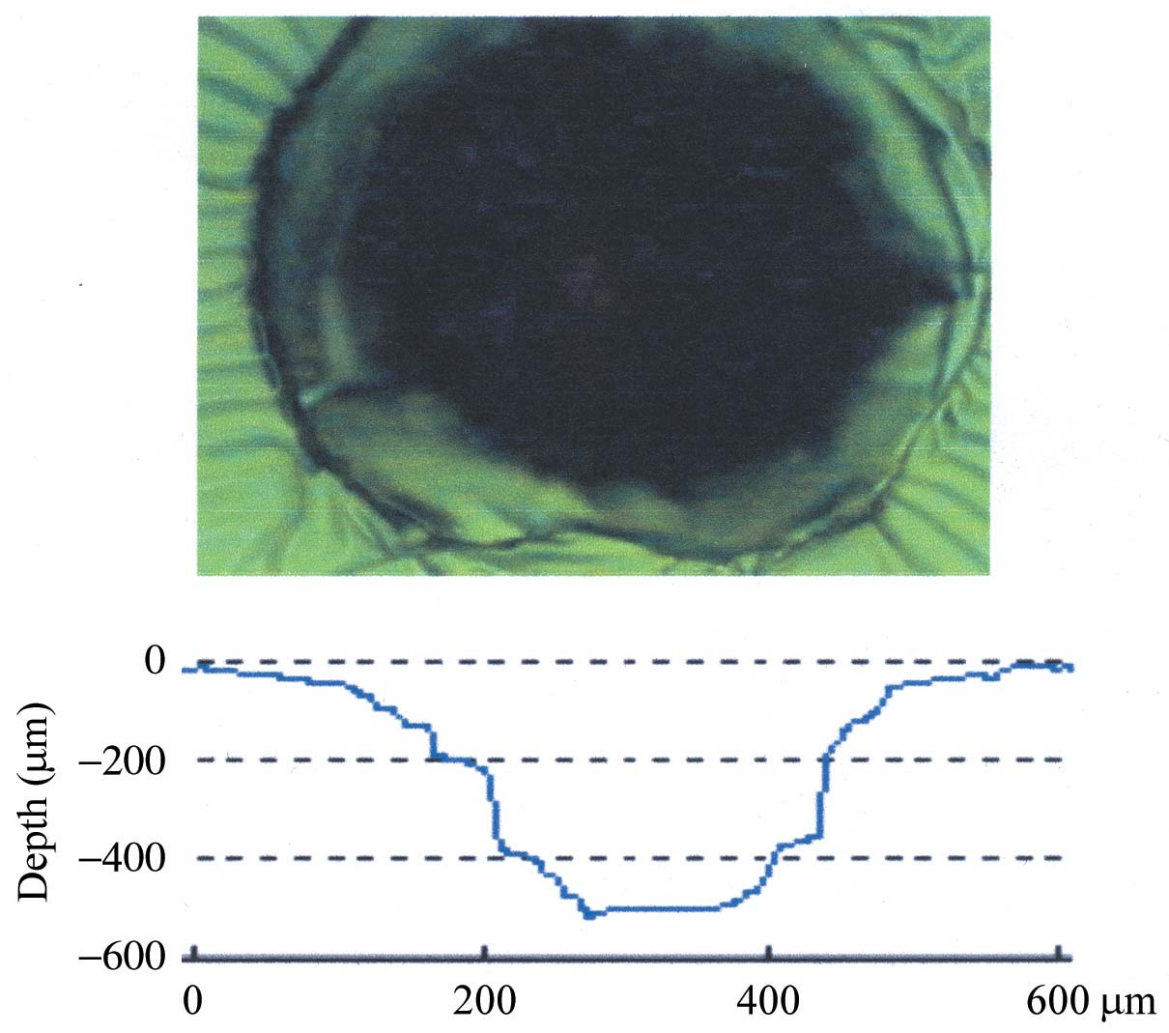
Top view of the hole formed by SR etching using XeF_2_ gas (0.5 torr) on the PDMS substrate surface observed by optical microscope. The SR beam current and the etching time were 150 mA and 10 min. The photoemission current measured by Pt detector was ∼312 µA. The measured profile of the hole is also given.

**Figure 5:**
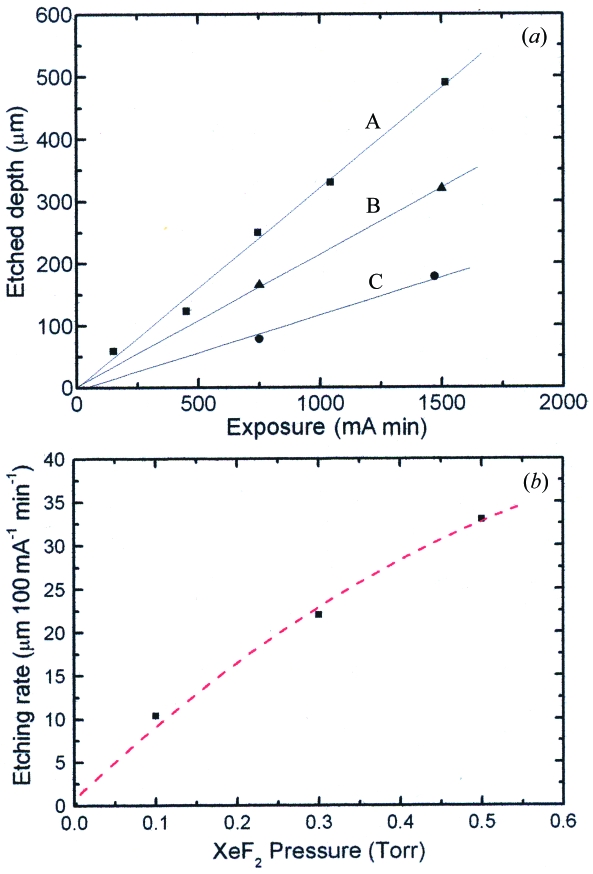
Observed dependence of the etched depth on SR exposure (*a*), and etching rate on the XeF_2_ pressure (*b*). A, B and C are measured at a XeF_2_ pressure of 0.5, 0.3 and 0.1 torr, respectively. The photoemission current measured by the Pt detector was ∼55 µA.

Fig. 4 ▶ shows the top view of the etched pattern on the PDMS surface under the condition of 0.5 torr XeF_2_ gas pressure, 150 mA ring current (photoemission current of the Pt plate was ∼312 µA) and 10 min irradiation time. As discussed later in detail, the carbon contamination was easily induced, especially when the vacuum pumping of the etching chamber was not sufficient. To reduce the contamination, the etching chamber should be pumped for 2–3 h before etching. Carbon contamination was more enhanced by the higher beam intensity. The dependence of the etching rate and etched depth on the irradiation dose and that on the XeF_2_ gas pressure measured under the same conditions are shown in Figs. 5(*a*) and 5(*b*) ▶, respectively. These data show that a high-speed, area-selective and anisotropic etching is realised for PDMS by the SR etching using XeF_2_ as an etching gas, although some rugged structures are observed surrounding the etched hole which are probably due to the heating effect of the SR beam. Note that the PDMS was not etched by XeF_2_ gas without SR irradiation.

**Figure 6:**
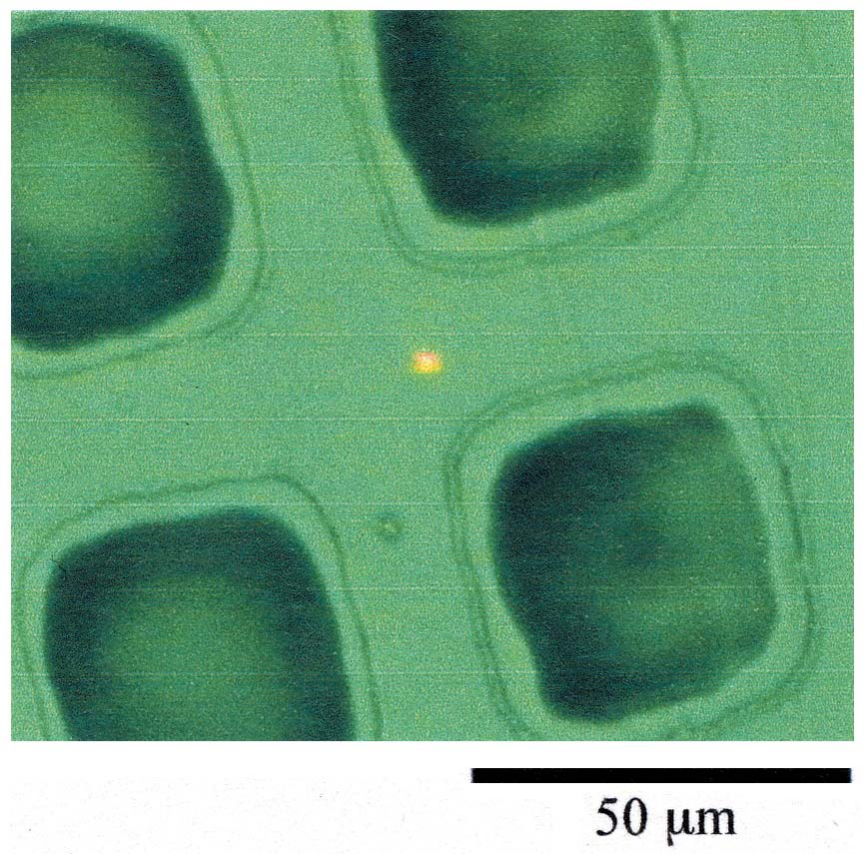
Optical microscope picture of the pattern formed on the PDMS substrate surface by 10 min of SR etching using the copper mesh with 400 wires per inch as a contact mask with the conditions of 0.4 torr XeF_2_ gas and 110 mA ring current. The etching chamber was evacuated for about 2 h before etching. The SR beam was attenuated to about 13% by the slit and the measured photoemission current was 30 µA. The measured depths of the holes formed by the etching was ∼40 µm.

**Figure 7:**
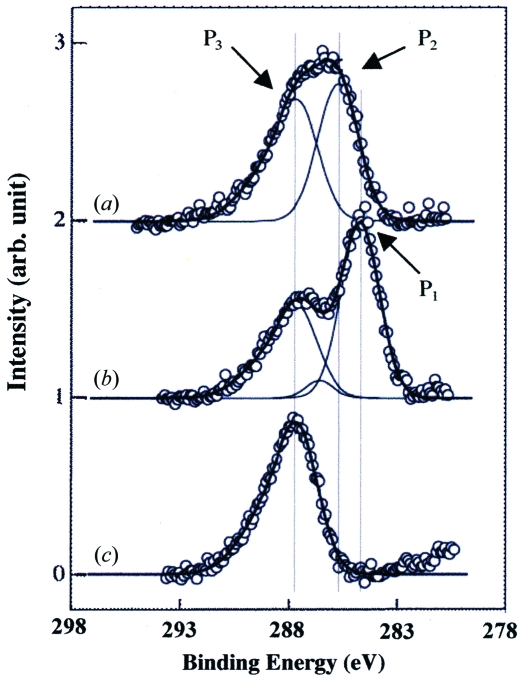
C 1*s* X-ray photoelectron spectroscopy (XPS) data. (*a*) The PDMS surface after 10 min etching by 0.2 torr XeF_2_ gas and 200 mA ring current. The SR beam was attenuated to about 13% by the slit. The etching chamber was evacuated for about 3 h before etching. The XPS was measured using an Au thin film covering the non-etched area of the PDMS surface. (*b*) The surface of PDMS without etching. (*c*) Au thin film used to cover the non-etched area of the PDMS surface. Peaks P1 (284.7 eV), P2 (285.7 eV) and P3 (287.7 eV) are assigned to carbon contained in the bulk PDMS, the hydrogen-containing carbon material (C_*x*_H_*y*_) deposited by the etching, and the carbon contamination on the Au thin-film surface, respectively.

In order to avoid the damage due to the SR beam heating effect, we tried etching with a lower SR beam intensity by using a slit set close to the centre of the beamline. The photoemission current at the sample position was about 30 µA at a ring current of 110 mA, which corresponds to about 10% of the intensity in the case shown in Fig. 4 ▶. The PDMS surface was masked by a copper mesh, whose thickness, window width and bar width were 20 µm, 30 µm and 33 µm, respectively. The PDMS surface shown in Fig. 6 ▶ was formed by SR etching through the mask at an XeF_2_ pressure of 0.4 torr and an etching time of 10 min. The depth of the etched hole was ∼40 µm. A similar result was obtained under the condition of 0.2 torr and ring current of 200 mA. After removal of the etching mask, each etched hole was found to have a size of 35 µm square at the bottom and 45 µm square at the top. The difference between the mask pattern and the etched pattern was explained by diffraction effects and an uncontrollable gap between the mask and the PDMS sample surface (Roth *et al.*, 2008 ▶). In particular, the side etching (∼20 µm depth) observed at the pattern edge (top of the hole) may be due to the secondary electrons, scattered photons and fluoride ions or metastable atoms entering the gap between the mesh and the PDMS surface. The carbon contamination was easily induced, especially when the vacuum pumping of the etching chamber was insufficient (data not shown). So we examined the etching effect of the PDMS by X-ray photoelectron spectroscopy, as shown in Fig. 7 ▶. The etching sample was prepared as follows. First, the etching chamber was pumped using a turbomolecular pump (not shown in Fig. 3 ▶) for 4 h. Then the etching was carried out for 10 min by 0.2 torr XeF_2_ gas and 200 mA ring current. The SR beam was attenuated to about 13% by the slit. The PDMS surface other than the irradiated area was covered by a gold thin film which was cleaned using piranha solution, H_2_SO_4_ + H_2_O_2_ (4:1), at 363 K for 10 min. C 1*s* spectra of the PDMS samples after etching, before etching and the gold thin film only are shown in Figs. 7(*a*), 7(*b*) and 7(*c*) ▶, respectively. Before etching, two features were observed in the C 1*s* region; one, at 284.7 eV, is assigned to C of PDMS (Roth *et al.*, 2008 ▶) and another, at 287.7 eV, to the carbon contamination, as shown in the spectrum of the gold thin film (see Fig. 7*c* ▶).

After etching, a new peak appeared at 285.7 eV, which is assigned to the hydrogen-containing carbon material (C_*x*_H_*x*_) (Paál & Schlögl, 2009 ▶). It was found that the hydrogen-containing carbon material would be dramatically reduced when the pressure in the etching chamber was lower than 10^−2^ torr. Under our experimental conditions, it was necessary to pump for at least 2–3 h to obtain a vacuum higher than 10^−2^ torr in the gas supply system shown in Fig. 3 ▶. It must be remarked that the 285.7 eV C 1*s* peak assigned to the hydrogen-containing carbon material was observed on the etched PDMS surface even when the black material almost disappeared by the sufficiently long-time pumping. From the fact that the deposition of the hydrogen-containing carbon material is sensitive to the base pressure of the chamber, it is considered that the removal of carbon contained in PDMS and the carbon deposition are competing during etching. Although the detailed mechanism has not been investigated in this experiment, this phenomenon, which is not observed in the SR etching of SiO_2_, is considered to be unique in PDMS which contains carbon as one of its main components.

Although we do not discuss the etching mechanisms in this work, the high spatial resolution, anisotropic and high-speed etching at room temperature indicates that the etching is induced by the electronic excitation of PDMS (Urisu & Kyuragi, 1987 ▶; Streller *et al.*, 1998 ▶; Akazawa *et al.*, 1991 ▶). The extremely high etching rate, 40–50 µm (10 min)^−1^, suggests that SR etching using XeF_2_ gas provides a new microfabrication technology for thick PDMS membrane which can open new applications such as the formation of three-dimensional microfluidic circuits.

## 4. Conclusions

A high-speed SR-etching beamline using XeF_2_ as a reaction gas has been constructed and high-spatial-resolution, area-selective and anisotropic etching of the elastic material PDMS has been demonstrated for the first time. An extremely high etching rate, 40–50 µm (10 min)^−1^, is realised, and this result indicates that SR etching using XeF_2_ gas provides a new microfabrication technology for thick PDMS membrane. Sufficient vacuum pumping of residual gases in the etching chamber is important to reduce the deposition of the hydrogen-containing carbon material, which hinders the etching. This may be a unique point of PDMS etching in which carbon is one of the main components.
